# Dietary Protein Intake Patterns and Inadequate Protein Intake in Older Adults from Four Countries

**DOI:** 10.3390/nu12103156

**Published:** 2020-10-16

**Authors:** Alejandro Gaytán-González, María de Jesús Ocampo-Alfaro, Francisco Torres-Naranjo, Roberto Gabriel González-Mendoza, Martha Gil-Barreiro, Maritza Arroniz-Rivera, Juan R. López-Taylor

**Affiliations:** 1 Institute of Applied Sciences for Physical Activity and Sport, Department of Human Movement Sciences, Education, Sport, Recreation, and Dance, University Health Sciences Center, University of Guadalajara, Guadalajara 44430, Mexico; dr.francisco.torres@icloud.com (F.T.-N.); roberto.gonzalez@academicos.udg.mx (R.G.G.-M.); taylor@cucs.udg.mx (J.R.L.-T.); 2 Department of Human Reproduction, Infantile Growth, and Development, University Health Sciences Center, University of Guadalajara, Guadalajara 44280, Mexico; 3 Geriatrics Department, Western General Hospital, Zapopan 45170, Mexico; mocampo1@prodigy.net.mx (M.d.J.O.-A.); marthagilbarreiro@yahoo.com.mx (M.G.-B.); marroniz.maritza@gmail.com (M.A.-R.); 4 Center of Body Composition and Bone Research, Guadalajara 44600, Mexico

**Keywords:** breakfast, meals, older adults, protein intake

## Abstract

Recent interest in protein intake per meal is observed in studies that have reported the protein intake patterns in different countries; however, comparisons of these data are lacking. We aimed to compare protein intake patterns and the percentage of inadequate protein intake (IPI) per day and meal in older adults from different countries. We acquired data of protein intake in older adults from four countries (Mexico, United States of America, Germany, and United Kingdom). We compared protein intake (per day and meal), IPI per day and meal, and the number of meals with an adequate protein content among countries. The IPI per day significantly differed among countries for <0.8 and <1.0 (both *p* < 0.001), but not for <1.2 g/kg/d (*p* = 0.135). IPI per meal (<30 g/meal) did not differ among countries at breakfast (*p* = 0.287) and lunch (*p* = 0.076) but did differ at dinner (*p* < 0.001). Conversely, IPI per meal (<0.4 g/kg/meal) significantly differed among countries at breakfast, lunch, and dinner (all *p* < 0.001). The percentage of participants that ate ≥30 g/meal or ≥0.4 g/kg/meal at zero, one, and two or three meals per day significantly differed among countries (all *p* < 0.05). IPI at breakfast and lunch (<30 g/meal) was a common trait in the analyzed samples and might represent an opportunity for nutritional interventions in older adults in different countries.

## 1. Introduction

The ageing process in humans encompasses many changes that ultimately lead to undesirable clinical conditions like a higher fat deposition, osteoporosis, sarcopenia, frailty, and physical disability [1,2]. Current research on ageing focuses on decreasing the risk of developing the previously mentioned conditions and designing effective interventions to improve the patient’s health [3,4,5].

Regarding sarcopenia, frailty, and physical disability, protein intake is one of the several factors linked to these clinical conditions [6]. A growing body of evidence suggests that daily protein intake above the recommended dietary allowance (R.D.A.) of 0.8 g/kg/d is associated with better physical performance, maintenance or even an increase in muscle mass, and decreased risk of physical disability. Therefore, it has been suggested to set the protein recommendation to a higher dose of 1.0–1.2 g/kg/d [7,8,9,10].

Additionally, it seems that other traits, like protein intake per meal and protein distribution, should be considered along with daily protein intake [11,12]. Nonetheless, the evidence is equivocal; other studies suggest that the protein intake pattern might not be an important variable to consider in terms of skeletal muscle-related outcomes (e.g., strength and functionality) [12,13,14]. The studies supporting the importance of protein distribution in older adults suggest that the consumption of 30 g of protein per meal or 0.4 g/kg per meal are associated with a higher skeletal muscle mass and strength and maximally stimulates muscle protein synthesis, respectively [15,16]. In this regard, some studies suggested that inadequate protein intake (<30 g/meal or <0.4 g/kg/meal) at specific meals might be a risk factor to consider as it is associated to a lower skeletal muscle mass, muscle strength, and functionality [17,18,19]. On the other hand, the number of meals that reach a protein content ≥30 g/meal or ≥0.4 g/kg/meal appears to be a protective factor as they are associated with a higher skeletal muscle mass, muscle strength, and lower physical disability [15,20,21]. Therefore, determining the percentage of older adults for both indicators might help visualize the magnitude of these two possible risk and protective factors.

Several studies have focused their attention on analyzing dietary protein intake patterns in older adults in different countries [14,19,20,21,22,23,24,25,26,27]. Nonetheless, to the best of our knowledge, direct comparisons of protein intake patterns among countries are lacking [28]. Similarly, the comparison of the percentage of older adults that did not eat enough protein per day (i.e., 0.8, 1.0, and 1.2 g/kg/d) or per meal (30 g/meal; 0.4 g/kg/meal) among countries is missing. These comparisons among countries might serve as a starting point to understand the magnitude of this situation, because comparisons would help us to determine if these different samples share common issues, allowing us to identify protein-eating patterns to be improved.

Therefore, this exploratory study aimed to (1) compare dietary protein intake patterns among older adults from four countries, (2) report and compare data of inadequate protein intake per day and per meal among older adults from four countries, and (3) analyze if these comparisons would yield similar results when the analysis was separated by sex. We hypothesized that dietary protein intake patterns would be different, but inadequate protein intake would be similar among countries. Likewise, we hypothesized that the comparisons among countries separated by sex would yield different patterns (e.g., if protein at breakfast differs among countries in women but not in men) and that the whole sample pattern would be the same in women but not in men.

## 2. Materials and Methods

### 2.1. Study Design and Data Acquisition

This is an exploratory analysis carried out with data from previously published articles where authors reported protein intake per day and meal in adults aged ≥60 years. Corresponding authors were contacted to gather demographic and protein intake data. We acquired data from two studies of two countries (Germany [14] and the United Kingdom [U.K.] [24]), from the National Health and Nutrition Examination Survey (NHANES) 2015–2016 publicly available database representing the United States of America (U.S.A.) [29], and data from our previous work in Mexico [18,20], all with cross-sectional designs.

To analyze the NHANES sample, we included data from participants with the following characteristics: (1) aged ≥60 year; (2) they were born in the U.S.A.; (3) reported an energy intake ≥600 and ≤4000 kcal/day; and (4) had complete data for age, height, and body mass. From these records (*n* = 1039, 50% women), we randomly selected 200 subjects (100 per sex to keep the sex proportion) to decrease the differences in sample size among groups. There were no significant differences between included and nonincluded subjects (*n* = 839) for age (*p* = 0.81), body mass (*p* = 0.55), height (*p* = 0.38), BMI (*p* = 0.98), nor total protein intake per day (*p* = 0.15). This sample was not weighted according to the NHANES complex study design as the other studies did not follow the same sampling design.

All studies independently coded the three main meals (i.e., breakfast, lunch, and dinner), and reported that they obtained participants’ written informed consent and ethical approval from their local institution before any assessment. Table 1 shows an overview of the included samples. 

### 2.2. Protein Intake Variables

When studies reported two or more days of dietary assessment, we averaged the protein intake per day and per meal, and we used these averages for further analysis. We calculated relative protein intake per day (g/kg body mass/d) and per meal (g/kg body mass/meal), meal contribution to total daily protein (%), and protein distribution coefficient of variation in addition to the absolute protein intake per day (g/d) and per meal (g/meal).

*Meal contribution* to total protein was calculated as:

(1) Meal contributrion = PM/TP × 100,

where *PM* is the protein reported for any given meal (g) and *TP* is the total daily protein intake (g).

The protein distribution coefficient of variation (*PDCV*) was calculated as:

(2) PDCV = SDP/MP,

where *SDP* is the standard deviation of the three main meals and *MP* is the mean protein intake for the three main meals.

### 2.3. Inadequate Protein Intake

Inadequate protein intake (IPI) was considered as any protein consumption <0.8 (IPID-0.8), <1.0 (IPID-1.0), and <1.2 (IPID-1.2) g/kg/d [7,8] or <30 g/meal (IPIM-30) and <0.4 g/kg/meal (IPIM-0.4) [15,16]. We reported IPIM-30 and IPIM-0.4 for each main meal. We also counted the number of meals per day (coded as zero [0M], one [1M], and two or three meals [+2M]) with ≥30 g protein and ≥0.4 g protein/kg each.

### 2.4. Statistics

Continuous data were expressed in mean ± standard deviation, while categorical and ordinal data in frequencies, percentages, and 95% confidence intervals. To compare the continuous variables among countries, we performed one-way ANOVAs with Scheffe test as post hoc when Levene’s test suggested homogeneity of variances (*p* > 0.05). Otherwise, one-way ANOVAs with Welch’s correction and Dunnett T3 test as post hoc were performed (Microsoft^®^ Excel^®^ 2013, Microsoft Corporation, Redmond, WA, USA). To compare the categorical and ordinal variables among countries, we used the χ^2^ test of independence (GraphPad Prism 7.05, GraphPad Software Inc., La Jolla, CA, USA) and multiple *t*-tests for proportions with Bonferroni correction as post hoc (Statistics calculator v4.0, StatPac, Northfield, MN, USA). All comparisons were deemed significant at a *p*-value ≤ 0.05. Effect sizes were calculated for ANOVAs (omega squared, ω^2^) and χ^2^ tests of independence (phi statistic, *φ*). Both effect size statistics are dimensionless and range between 0 and 1. An effect size was considered small, medium, or large if ω^2^ reached 0.01, 0.06, or 0.14, respectively; the respective values for *φ* were 0.1, 0.3, 0.5 [30,31]. Effect sizes below the “small” cut point were considered trivial [32]. The difference between the highest mean and the lowest mean was reported along with the effect size. The comparisons among countries were carried out for the whole sample and separated by sex.

## 3. Results

### 3.1. Demographic Data

There were significant differences among countries for all demographic data. Mexico showed the highest women proportion while Germany the lowest (25% difference, small effect). Age, body mass, and height comparisons showed a large effect. The oldest participants came from Mexico, Germany, and U.K. in contrast to those in U.S.A. (8-year difference). The heaviest participants belonged to U.S.A. (20 kg difference vs. Mexico), and U.S.A. and Germany showed the tallest participants (13 cm difference vs. Mexico). The B.M.I. comparison showed a medium effect with U.S.A. having the heaviest participants (3.3 units difference vs. Germany) (Table 2). A similar pattern was observed in women (Table S1) and men (Table S2).

### 3.2. Protein Intake

For absolute protein intake (g), U.K., U.S.A., and Germany showed the highest protein consumption per day (19 g difference vs. Mexico, medium effect). Mexico, U.S.A., and Germany showed the highest intake at breakfast (3 g difference vs. U.K., trivial effect). In comparison, U.K. and U.S.A. showed the highest intake at dinner (20 g difference vs. Mexico, large effect). There were no significant differences among countries for protein intake at lunch (Table 2).

For relative protein intake (g/kg), U.K. showed the highest intake per day (0.22 g/kg difference vs. U.S.A., small effect). Mexico showed the highest intake at breakfast (0.09 g/kg difference vs. U.S.A., medium effect). U.K. and Mexico showed the highest intake at lunch (0.15 g/kg difference vs. U.S.A., medium effect). U.K. showed the highest intake at dinner (0.29 g/kg difference vs. Mexico, large effect) (Table 2).

Analyzing the percentage of meal contribution to total protein intake, Germany, and Mexico ate most of their daily protein at lunch, whereas U.S.A. and U.K. did it at dinner. Mexico showed the highest breakfast (13% difference vs. U.K., medium effect) and lunch contribution (12% difference vs. U.S.A., medium effect), whereas U.S.A. and U.K. showed the highest dinner contribution (19% difference vs. Mexico, large effect). In terms of the PDCV, Germany showed the evenest protein distribution (−0.17 units difference vs. U.K., small effect) (Table 2). The comparisons divided by sex showed a very similar pattern to that observed of the whole sample, with slight differences in which countries differ from one another (Tables S1 and S2). However, it is remarkable that there were no significant differences among countries at breakfast and lunch (g) nor per day and breakfast (g/kg) in men (Table S2).

### 3.3. Inadequate Protein Intake per Day

**Figure 1:**
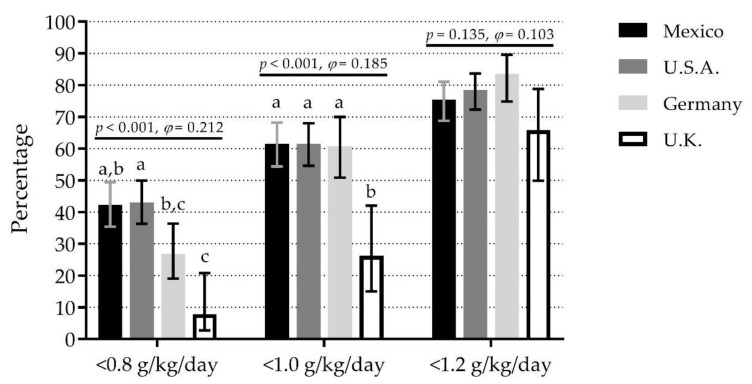
Comparison of inadequate protein intake per day with different cut points among four countries. Bars represent the percentage of inadequate protein intake per day; whiskers represent 95% confidence intervals. *p*-values and *φ* statistic are for comparisons among countries within cut points (χ^2^ test of independence). Bars not sharing a similar letter (a, b, c) denote significant differences (*p* ≤ 0.05) among countries within cut points (*t*-test for proportions with Bonferroni correction). g/kg/day: grams of protein per kilogram of body mass per day; U.K.: United Kingdom; U.S.A.: United States of America. Detailed data can be found in Table S3.

There were significant differences among countries for IPID-0.8, IPID-1.0, but not IPID-1.2. For IPID-0.8, the highest percentage was observed in U.S.A. (43.0%) and Mexico (42.2%) (35% difference vs. U.K., small effect). For IPID-1.0, U.S.A. (61.5%), Mexico (61.5%), and Germany (60.8%) showed the highest percentages (35% difference vs. U.K., small effect). For IPID-1.2, percentages ranged from 65.8% in U.K. to 83.5% in Germany (Figure 1). A similar pattern was observed in women. However, in men, there were no significant differences among countries for any cut point (Figure S1).

### 3.4. Inadequate Protein Intake per Meal

**Figure 2:**
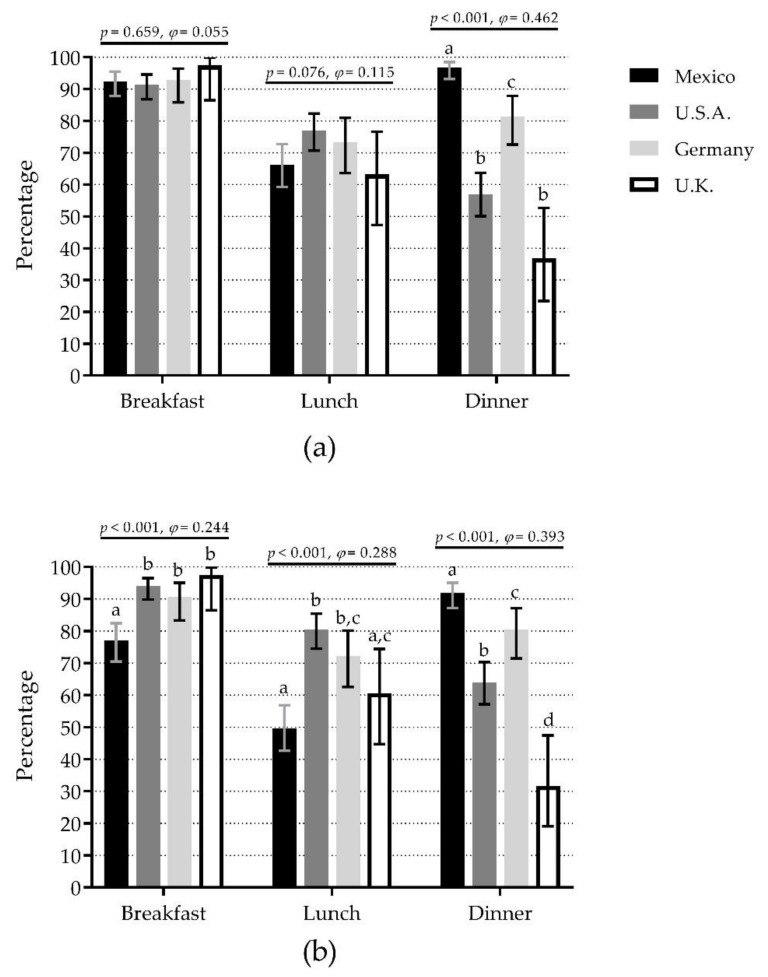
Comparison of inadequate protein intake per meal (breakfast, lunch, and dinner) among four countries depending on the protein content at each meal as <30 g/meal (**a**) or <0.4 g/kg body mass/meal (**b**). Bars represent the percentage of inadequate protein intake per meal; whiskers represent 95% confidence intervals. *p*-values and *φ* statistic are for comparisons among countries within meals (χ^2^ test of independence). Bars not sharing a similar letter (a, b, c, d) denote significant differences (*p* ≤ 0.05) among countries within meals (*t*-test for proportions with Bonferroni correction). U.K.: United Kingdom; U.S.A.: United States of America. Detailed data can be found in Table S3.

For IPIM-30, there were no significant differences among countries at breakfast (range 91.5% in U.S.A. to 97.4% in U.K.) and lunch (range 63.2% in U.K. to 77.0% in U.S.A.). However, there were significant differences at dinner, where Mexico (96.8%) showed the highest percentage (60% difference vs. U.K., medium effect) (Figure 2a). The pattern was very similar when comparisons were separated by sex (Figure S2a,b).

For IPIM-0.4, there were significant differences among countries for the three meals. At breakfast, U.K. (97.4%), U.S.A. (94.0%), and Germany (90.7%) showed the highest percentages (20% difference vs. Mexico, small effect). At lunch, U.S.A. (80.5%) and Germany (72.2%) showed the highest percentages (31% difference vs. Mexico, small effect). At dinner, Mexico (92.0%) showed the highest percentage (61% difference vs. U.K., medium effect) (Figure 2b). The pattern was very similar when comparisons were separated by sex (Figure S2c,d).

### 3.5. Number of Meals per Day with Adequate Protein Content

**Figure 3:**
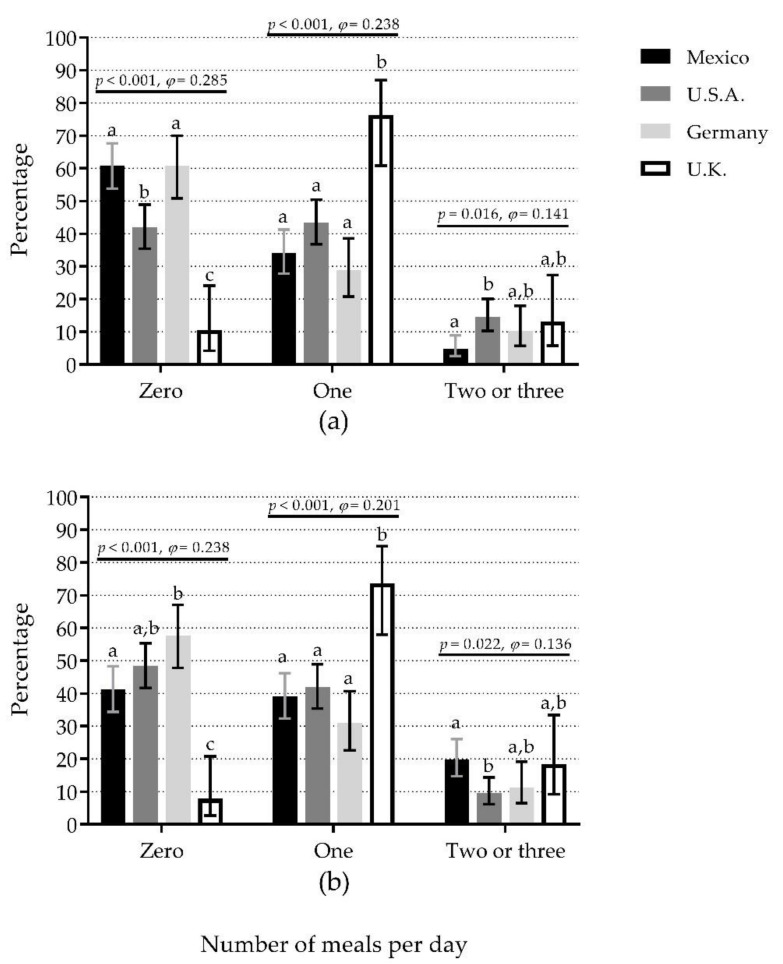
The number of meals per day containing ≥30 g protein (**a**) or ≥0.4 g protein/kg body mass (**b**) compared among countries. Bars represent the percentage of participants that reported the number of meals per day (zero, one, and two or three) with the mentioned protein content; whiskers represent 95% confidence intervals. *p*-values and *φ* statistic are for comparisons among countries within the number of meals (χ^2^ test of independence). Bars not sharing a similar letter (a, b, c) denote significant differences (*p* ≤ 0.05) among countries within the number of meals (*t*-test for proportions with Bonferroni correction). U.K.: United Kingdom; U.S.A.: United States of America. Detailed data can be found in Table S3.

When the ≥30 g protein/meal criterion was used, Mexico (61.0%) and Germany (60.8%) showed the highest percentages of 0M (50% difference vs. U.K., small effect), U.K. (76.3%) showed the highest percentage of 1M (48% difference vs. Germany, small effect), whereas U.S.A. (14.5%) showed the highest percentage of +2M (10% difference vs. Mexico, small effect) (Figure 3a). The pattern was similar when comparisons were separated by sex, except there were no significant differences among countries for +2M for either sex (Figure S3a,b).

When the ≥0.4 g protein/kg/meal criterion was used, Germany (57.7%) and U.S.A. (48.5%) showed the highest percentages of 0M (50% difference vs. U.K., small effect), whereas U.K. (73.7%) showed the highest percentage of 1M (42% difference vs. Germany, small effect). In contrast, Mexico (19.8%) showed the highest percentage of +2M (10% difference vs. U.S.A., small effect) (Figure 3b). The comparisons separated by sex showed a different pattern to that observed in the whole sample. In women, there were significant differences among countries for 0M and 1M, but not for +2M groups (Figure S3c). In men, there were significant differences among countries for 0M, but not for 1M and +2M groups (Figure S3d).

### 3.6. IPI Combined Results

When data from the four samples were combined (*n* = 522), we calculated the percentage of IPID-0.8, IPID-1.0, IPID-1.2, IPIM-30, IPIM-0.4, and the number of meals with an adequate protein content (Table 3). Briefly, we observed that there was about a 20% increase in the percentage of IPI per day every time when the cut point was increased. For both IPIM-30 and IPIM-0.4, breakfast was the meal with the highest percentage, followed by dinner and lunch. For both ≥30 g/meal and ≥0.4 g/kg/meal criteria, eating 0M was the most condition, followed by 1M and +2M.

## 4. Discussion

To the best of our knowledge, this is the first study that compares dietary protein intake patterns and the percentage of IPI per day and per meal among countries. While there are studies where dietary protein intake patterns were reported, direct evidence about their differences was lacking. This study demonstrates that some dietary protein intake patterns in older adults significantly differed among countries, but they shared traits in IPI. For instance, three countries showed a similar percentage of IPID-1.0 (>60%) and IPID-1.2 (>75%) (Figure 1). These high percentages of IPI per day might represent a higher risk of lower physical functioning [9,10,33] and developing frailty [34,35].

Furthermore, the main finding of this study was that participants from all the analyzed samples reported a high percentage of IPIM-30 at breakfast (>90%) and lunch (>60%) (Figure 2a). This is relevant due to IPI at breakfast might also be attributable to breakfast skipping [36]. Although breakfast skipping is less studied in older adults, evidence in their younger counterparts offers insight about what to expect. For example, breakfast skipping was associated with lower skeletal muscle mass in young adults [37], which seems feasible in older adults because meal skipping might lead to malnutrition [38] and skeletal muscle loss [39]. Additionally, breakfast skipping is associated with a higher risk of developing type 2 diabetes in adults of different ages (18–83 year) [40]. Moreover, breakfast has been previously reported as a low-energy containing meal [36]. For instance, Huseinovic et al. [28] reported that breakfast composed the lowest contribution to the daily energy intake in different European countries, which we speculate might include IPIM-30. On the other hand, insufficient protein intake at lunch is also associated with lower functionality and muscle mass decline [18,19]. Both eating occasions might represent an opportunity for nutritional interventions in older adults in many countries; however, breakfast appears to be a priority due to its larger percentage of inadequate protein intake.

We observed that most differences among countries could be attributable to women because the pattern was very similar to that observed in the whole sample. However, while it appears to be a sex-derived pattern in some variables (e.g., protein at breakfast and lunch), it is important to highlight that there were more women than men in this analysis. Therefore, men could be underrepresented and different patterns attributable to different sample sizes. Further research among countries with larger men samples is recommended.

As previously mentioned, the evidence comparing eating patterns among countries is scarce, and the available studies are focused on eating occasions, meal frequency, and meal energy content, rather than protein intake per meal [28,36,41]. Therefore, it is difficult to draw comparisons with the previous evidence. However, we can see that our results are supported by drawing inferences from other studies; i.e., IPI per day may differ among countries [42,43], but IPI at breakfast appears to be high [43,44]. Although an “all countries” comparison might not be reliable, we believe it would be of interest to compare countries sharing a geographical area (e.g., North America, South America, and Western Europe) and among states/provinces within countries. It would lead to data about the magnitude of the problem in specific regions to design concrete strategies to overcome this problem.

Another important finding is that about 90%, 70%, and 74% of the participants did not eat enough protein at breakfast, lunch, and dinner, respectively (Table 3). Therefore, there is interest in strategies to increase total dietary protein intake [4,5,45]. We [20] and others [15,21,46] have previously reported that reaching an adequate protein content per meal (i.e., ≥30 g) is significantly associated with higher daily protein intake and lower proportion of IPI per day (<1.2 g/kg/d). However, this practice is not usual. We reported here that only about 15% of participants ate two or three meals with adequate protein content (Table 3). This low proportion of compliance might be attributable to the lack of appetite and some chewing difficulties commonly observed in older adults [5,6,47], thus making it challenging to reach this protein amount with simple foods [47]. Some strategies, like mincing meat [48] and food enrichment with protein [46], may help overcome the difficulties mentioned earlier. On the other hand, about 45% of the participants did not eat enough protein at any meal (Table 3), which may be a concern because their muscle protein synthesis might not be stimulated to its maximum level at any meal [11]. This low protein intake might lead to a negative protein balance, muscle loss, and possibly accelerating the functional decline in the elderly [49,50].

In this study, the combined data suggested that about 40%, 60%, and 80% of the participants showed IPI per day for the <0.8, <1.0, and <1.2 g/kg criteria (Table 3). These results are similar to those recently reported by Hengeveld et al. [51]. In their meta-analysis, they found that when protein intake was divided by the actual body weight (g/kg of actual body weight/day), the IPI was 29.1, 54.3, and 75.7%, respectively. Nevertheless, when they used the adjusted body weight (g/kg adjusted body weight/day), the percentages of IPI were lower, 21.5%, 46.7%, and 70.8%, respectively. It is important to note that Hengeveld et al. analyzed data from adults aged ≥55 year, belonging to large cohorts (*n* = 410 to 2660) from different countries (U.S.A., the Netherlands, U.K., Canada, Finland, and Italy) and that they performed complex statistics to came up with the previously mentioned percentages. However, despite these differences in comparison to our study, they showed similar results when the protein intake was reported with the actual body weight for the 1.0 and 1.2 g/kg/d criteria. Nonetheless, the authors suggest using the adjusted body weight to decrease the chances of over- or underestimation [25,51].

It is important to consider this study’s limitations. First, meal identification varied among studies (participant-identified [18,29]; time of the day [24]; time of the day and meal composition [14]); therefore, it impedes us to analyze the specific hour when occurred. Thus, meals could be close to one another in the time of the day but were categorized differently due to cultural traits [36]. Hence, it is difficult to fully conclude differences among meals, at least at their respective timing (i.e., breakfast, lunch, and dinner) [36]. This is one of the most important limitations to consider when translating results regarding the timing of IPI and functionality in older adults [17,18]. Consequently, the number of meals per day that reach adequate protein content might offer a better approach than meal timing labelling. This number has shown significant associations with higher muscle mass, strength, and lower risk of physical disability, even in samples that showed different protein eating patterns (e.g., eating more protein at dinner [15] vs. lunch [20]). Thus, it is less likely to be affected by differences in meal labelling [36].

Second, we did not analyze snacks because its categorization is more complex than with the main meals [36]. Nevertheless, further studies should include them because some protein-rich foods (e.g., dairy) might be commonly eaten at snacks [22,43]. In addition, snacks might represent as much energy as a main meal when merged [28], and possibly, the same is true with protein intake.

Third, we had no data about the foods consumed (e.g., meat, dairy, and grains). Protein sources are important because animal protein has a higher anabolic effect than vegetable proteins; this effect is mainly attributable to higher amino acid availability in animal-based foods than those coming from plant-based foods [52]. Therefore, the inclusion of data of ingested foods and food intake patterns along the determination of IPI per day and per meal might help to understand their combined role on health-related variables and which foods are associated with a lower probability of inadequacy [19,53,54,55].

Fourth, there were different dietary assessment methods for all studies (Table 1), and each one addresses the information from a different approach [56,57]. Moreover, the results should be interpreted cautiously because recall methods are prone to underreporting when compared to doubly labelled water, and two studies relied on one- and 2-days dietary recalls [20,29], which may poorly represent the actual nutritional intake [58,59]. Additionally, the differences in the nutritional analysis due to different food databases must be considered. It is important to highlight that data reported in this study might differ from that in the original articles due to differences in how it was analyzed. For example, Gingrich et al. [14] calculated the PDCV for each day of the 7-day diary and then averaged them. Instead, we first averaged the 7-day protein intake, and then we calculated the PDCV. As mentioned in the methods section, all databases followed the same procedure to average protein intake and made comparisons possible.

Fifth, these results came from very localized samples and must not be deemed nationally representative (Table 1). Instead, these data should be considered a glance into how the dietary protein intake patterns are in these countries. In the case of NHANES, it is a nationally representative study; however, we analyzed a sample of the available cases (200 out of 1039), and data were not weighted according to the complex sampling design; therefore, it cannot be considered as nationally representative. Comparisons of data coming from nationally representative studies deserve further research [51]. Similarly, the combined data reported here should be regarded with caution for several reasons: (1) it came from very different samples (Table 1 and Table 2); (2) there are some studies reporting protein intake per meal that were not included here, and the lack of these data surely affect the reported percentages; and (3) the percentages were not weighted. A meta-analysis of the available evidence with weighted pooled prevalence would help to overcome this limitation. We hope this work stimulates the curiosity to carry out systematic reviews with meta-analysis in this area.

The recruitment period may be another limitation. Although the recruitment years were close to one another (about 3 years), this period may yield differences in dietary patterns within countries. Similarly, the recruitment method differed among studies (Table 1). While all participants were community-dwelling, there may be differences in sociodemographic (e.g., education and income) and health-related variables (e.g., functionality and diagnosed diseases) (not included here) that may explain the differences in the outcome variables instead of the geographical localization. The addition and comparison of these variables for further studies are recommended. Additionally, mean ages ranged from 71 to 79 year; this eight-year gap might have influenced the differences observed among countries as older adults tend to eat less protein [23,25]. Finally, the differences in sample sizes in some comparisons were considerable (38 vs. 200, min vs. max), affecting the statistical tests.

## 5. Conclusions

Inadequate protein intake at breakfast and lunch (<30 g/meal) was a common trait in the analyzed samples, even though most dietary protein intake patterns differed among them. Therefore, these two eating occasions might represent an opportunity for nutritional interventions in older adults in different countries. Similarly, the consumption of two or three meals per day with adequate protein content is less frequent. Further research is recommended to analyze if better dietary protein intake patterns match with better functionality or other health-related variables.

## Figures and Tables

**Table 1:** Description of studies’ main characteristics.

Author (Year)	City and Country	Year of Recruitment	Sample Size	Representativity	Setting	Recruitment	Food Assessment Tool
			**(W/M)**				
Gaytán-González (2020) [20]	Zapopan, Mexico	2017	187	Local	Community-dwelling	Users of a tertiary care hospital	One 24-h dietary recall
			(140/47)				
NHANES, 2015–2016 [29]	United States of America	2015–2016	200	National ‡	Community-dwelling	Random selection from the national census	Two nonconsecutive 24-h dietary recall
			(100/100)				
Gingrich (2017) [14]	Nürnberg, Germany	2016–2017	97	Local	Community-dwelling	Citizen registry	7-day food record
			(48/49)				
Cardon-Thomas (2018) [24]	Birmingham, United Kingdom	2014	38	Local	Community-dwelling	Volunteer databases	3-day food diary
			(26/12)				

NHANES: National Health and Nutrition Examination Survey; W/M: number of women and men. ‡ The complex sampling design of NHANES leads to nationally representative data; however, this is not the case for this study as the data were not weighted according to its sampling design and were composed of a smaller sample size (200 vs. 1039).

**Table 2:** Participants’ main characteristics and protein intake variables by country.

	Mexico	U.S.A.	Germany	United Kingdom	*p*-Value	Effect Size	
*n*	187	200	97	38			
Women (%)	74.9 a	50.0 b	49.5 b	68.4 a,b	<0.001	0.244	S
Age (year)	79 ± 8 a	71 ± 7 b	78 ± 3 a	78 ± 5 a	<0.001	0.226	L
Body mass (kg)	63.2 ± 13.1 a	83.8 ± 19.6 b	74.1 ± 14.0 c	68.0 ± 12.0 a,c	<0.001	0.238	L
Height (cm)	153.6 ± 9.1 a	166.6 ± 10.3 b	166.1 ± 9.2 b	- †	<0.001	0.297	L
BMI (kg/m^2^)	26.9 ± 5.7 a	30.1 ± 6.0 b	26.8 ± 4.0 a	- †	<0.001	0.074	M
Absolute protein intake (g)							
Day	57 ± 20 a	74 ± 28 b	70 ± 19 b	76 ± 12 b	<0.001	0.111	M
Breakfast	17 ± 8 a	16 ± 10 a,b	16 ± 9 a,b	14 ± 6 b	0.023	0.006	T
Lunch	25 ± 12	23 ± 14	24 ± 10	29 ± 14	0.098	0.009	T
Dinner	13 ± 8 a	30 ± 17 b	22 ± 11 c	33 ± 12 b	<0.001	0.275	L
Relative protein intake (g/kg)							
Day	0.93 ± 0.37 a	0.92 ± 0.38 a	0.97 ± 0.28 a	1.14 ± 0.25 b	<0.001	0.019	S
Breakfast	0.29 ± 0.16 a	0.20 ± 0.13 b	0.23 ± 0.12 b	0.21 ± 0.10 b	<0.001	0.077	M
Lunch	0.41 ± 0.22 a	0.28 ± 0.19 b	0.33 ± 0.14 c	0.43 ± 0.23 a,c	<0.001	0.082	M
Dinner	0.21 ± 0.14 a	0.37 ± 0.22 b	0.30 ± 0.14 c	0.50 ± 0.19 d	<0.001	0.196	L
Daily contribution (%)							
Breakfast	32 ± 14 a	22 ± 13 b,c	23 ± 10 b	19 ± 8 c	<0.001	0.126	M
Lunch	43 ± 15 a	31 ± 16 b	35 ± 12 b	38 ± 16 a,b	<0.001	0.114	M
Dinner	23 ± 12 a	40 ± 16 b	31 ± 11 c	44 ± 15 b	<0.001	0.268	L
PDCV	0.55 ± 0.26 a	0.59 ± 0.28 a	0.43 ± 0.24 b	0.60 ± 0.18 a	<0.001	0.044	S

† Data not obtained. BMI: Body Mass Index; g/kg: grams of protein per kilogram of body mass; L: large effect size; M: medium effect size; PDCV: protein distribution coefficient of variation (dimensionless); S: small effect size; T: trivial effect size; U.S.A.: United States of America. Countries not sharing a similar letter denote significant differences among them (*p* ≤ 0.05) within each variable.

**Table 3:** Combined data (*n* = 522) from the four samples analyzed.

	*n*	(%)	(95% CI)
IPID-0.8	194	(37.2)	(33.1–41.4)
IPID-1.0	307	(58.8)	(54.5–63.0)
IPID-1.2	404	(77.4)	(73.6–80.8)
IPIM-30			
Breakfast	483	(92.5)	(89.9–94.5)
Lunch	373	(71.5)	(67.4–75.2)
Dinner	388	(74.3)	(70.4–77.9)
IPIM-0.4			
Breakfast	457	(87.5)	(84.4–90.1)
Lunch	347	(66.5)	(62.3–70.4)
Dinner	390	(74.7)	(70.8–78.3)
Number of meals with ≥30 g/meal			
Zero	261	(50.0)	(45.7–54.3)
One	208	(39.8)	(35.7–44.1)
Two or three	53	(10.2)	(7.8–13.0)
Number of meals with ≥0.4 g/kg/meal			
Zero	233	(44.6)	(40.4–48.9)
One	215	(41.2)	(37.0–45.5)
Two or three	74	(14.2)	(11.4–17.4)

95% CI: 95% confidence intervals; IPID-0.8: inadequate protein intake per day (<0.8 g/kg/d); IPID-1.0: inadequate protein intake per day (<1.0 g/kg/d); IPID-1.2: inadequate protein intake per day (<1.2 g/kg/d); IPIM-30: inadequate protein intake per meal (<30 g/meal); IPIM-0.4: inadequate protein intake per meal (<0.4 g/kg/meal).

## Supplementary Materials

The following are available online at https://www.mdpi.com/2072-6643/12/10/3156/s1, Table S1: Women’s main characteristics and protein intake variables by country. Table S2: Men’s main characteristics and protein intake variables by country. Table S3: Detailed inadequate protein intake per day and meal in older adults from four countries. Figure S1: Comparison of inadequate protein intake per day with different cut points among four countries in women and men. Figure S2: Inadequate protein intake per meal (breakfast, lunch, and dinner) compared among four countries depending on the protein content for each meal as <30 g/meal or <0.4 g/kg body mass/meal in women and men. Figure S3: The number of meals per day containing ≥30 g protein or ≥0.4 g protein/kg body mass each, in women and men and compared among countries.
