# Supplementary material for: Dietary Protein Intake Patterns and Inadequate Protein Intake in Older Adults from Four Countries
Source: Nutrients. 2020 Oct 16;12(10):3156. doi: 10.3390/nu12103156 (PMC7602881; doi:10.3390/nu12103156)
Supplement: Supplementary file 1 [file nutrients-12-03156-s001.pdf]

Table S1. Women's main characteristics and protein intake variables by country.

	Mexico	U.S.A.	Germany	United Kingdom	*p*-value	Effect size	
**n**	**140**	**100**	**48**	**26**			
Age (y)	78 ±8 a	71 ±7 b	78 ±3 a	78 ±5 a	<0.001	0.184	L
Body mass (kg)	62.2 ±13.4 a	77.6 ±19 b	66.0 ±11.1 a	65.3 ±11.3 a	<0.001	0.162	L
Height (cm)	150.7 ±7.6 a	159.5 ±7.7 b	158.9 ±5.7 b	-†	<0.001	0.250	L
BMI (kg/m^2)	27.4 ±6.1 a	30.4 ±6.6 b	26.2 ±4.3 a	-†	<0.001	0.062	M
Absolute protein intake (g)							
Day	55 ±18 a	67 ±24 b,c	63 ±14 b	73 ±10 c	<0.001	0.089	M
Breakfast	17 ±8 a	13 ±8 b	15 ±8 a,b	13 ±5 a,b	0.001	0.040	S
Lunch	24 ±12	21 ±14	22 ±9	27 ±14	0.153	0.011	S
Dinner	12 ±8 a	28 ±15 b	18 ±8 c	33 ±11 b	<0.001	0.333	L
Relative protein intake (g/kg)							
Day	0.93 ±0.37 a	0.91 ±0.41 a	0.97 ±0.25 a	1.14 ±0.22 b	0.001	0.019	S
Breakfast	0.29 ±0.15 a	0.19 ±0.13 b	0.23 ±0.13 a,b	0.20 ±0.08 b	<0.001	0.096	M
Lunch	0.40 ±0.22 a	0.28 ±0.19 b	0.34 ±0.15 a,b	0.42 ±0.22 a	<0.001	0.062	M
Dinner	0.21 ±0.14 a	0.38 ±0.23 b	0.28 ±0.13 c	0.52 ±0.19 d	<0.001	0.236	L
Daily contribution (%)							
Breakfast	32 ±14 a	21 ±12 b,c	24 ±12 b	18 ±7 c	<0.001	0.158	L
Lunch	43 ±15 a	31 ±16 b	35 ±13 b	36 ±15 a,b	<0.001	0.099	M
Dinner	22 ±12 a	41 ±16 b	28 ±10 c	46 ±14 b	<0.001	0.323	L
PDCV	0.55 ±0.26 ab	0.62 ±0.27 a	0.44 ±0.22 b	0.60 ±0.19 a,b	0.002	0.038	S

† Data not obtained.

B.M.I.: Body mass index; g/kg: grams of protein per kilogram of body mass; L: Large effect size; M: Medium effect size; S: Small effect size; PDCV: Protein distribution coefficient of variation (dimensionless); U.S.A.: United States of America..

Countries not sharing a similar letter denote significant differences between them ($p \leq 0.05$) within each variable.

Table S2. Men's main characteristics and protein intake variables by country.

	Mexico	U.S.A.	Germany	United Kingdom	*p*-value	Effect size	
**n**	**47**	**100**	**49**	**12**			
Age (y)	80 ±7 a	71 ±7 b	78 ±3 a	76 ±5 a	<0.001	0.294	L
Body mass (kg)	66.1 ±12.1 a	90.0 ±18.2 b	81.9 ±11.9 c	73.9 ±11.8 a,c	<0.001	0.275	L
Height (cm)	161.9 ±7.9 a	173.7 ±7.1 b	173.1 ±5.9 b	-†	<0.001	0.326	L
BMI (kg/m^2)	25.1 ±3.9 a	29.7 ±5.2 b	27.3 ±3.7 c	-†	<0.001	0.139	M
Absolute protein intake (g)							
Day	61 ±24 a	82 ±29 b	77 ±21 b	81 ±15 a,b	<0.001	0.082	M
Breakfast	18 ±10	18 ±10	18 ±9	16 ±7	0.910	0.000	T
Lunch	28 ±14	24 ±15	26 ±10	32 ±16	0.322	0.010	S
Dinner	14 ±10 a	33 ±19 b	25 ±11 c	33 ±15 b,c	<0.001	0.184	L
Relative protein intake (g/kg)							
Day	0.95 ±0.39	0.93 ±0.34	0.96 ±0.30	1.14 ±0.30	0.244	0.005	T
Breakfast	0.28 ±0.16	0.21 ±0.13	0.22 ±0.11	0.23 ±0.12	0.063	0.035	S
Lunch	0.43 ±0.21 a	0.28 ±0.19 b	0.33 ±0.13 b	0.45 ±0.26 a,b	0.001	0.099	M
Dinner	0.21 ±0.15 a	0.37 ±0.20 b	0.32 ±0.15 b	0.45 ±0.21 b	<0.001	0.113	M
Daily contribution (%)							
Breakfast	30 ±11 a	23 ±13 b	23 ±9 b	21 ±10 a,b	0.003	0.053	S
Lunch	45 ±15 a	31 ±16 b	34 ±11 b	40 ±17 a,b	<0.001	0.120	M
Dinner	23 ±12 a	39 ±15 b	33 ±12 b	39 ±17 b	<0.001	0.165	L
PDCV	0.52 ±0.26 a,b	0.56 ±0.29 a	0.41 ±0.26 b	0.59 ±0.17 a,b	0.013	0.038	S

† Data not obtained.

B.M.I.: Body mass index; g/kg: grams of protein per kilogram of body mass; L: Large effect size; M: Medium effect size; PDCV: Protein distribution coefficient of variation (dimensionless); S: Small effect size; T: Trivial effect size; U.S.A.: United States of America.

Countries not sharing a similar letter denote significant differences between them ($p \leq 0.05$) within each variable.

Table S3. Detailed inadequate protein intake per day and meal in older adults from four countries.

	Mexico (n=187)			U.S.A. (n=200)			Germany (n=97)			United Kingdom (n=38)		
	n	%	95%CI	n	%	95%CI	n	%	95%CI	n	%	95%CI
Inadequate protein intake per day												
<0.8 g/kg/d	79	(42.2)	35.4 – 49.4	86	(43.0)	36.3 – 49.9	26	(26.8)	19.0 – 36.4	3	(7.9)	2.7 – 20.8
<1.0 g/kg/d	115	(61.5)	54.4 – 68.2	123	(61.5)	54.6 – 68.0	59	(60.8)	50.9 – 69.9	10	(26.3)	15.0 – 42.0
<1.2 g/kg/d	141	(75.4)	68.8 – 81.0	157	(78.5)	72.3 – 83.6	81	(83.5)	74.9 – 89.6	25	(65.8)	49.9 – 78.8
Inadequate protein intake per meal (<30 g/meal)												
Breakfast	173	(92.5)	87.8 – 95.5	183	(91.5)	86.8 – 94.6	90	(92.8)	85.8 – 96.5	37	(97.4)	86.5 – 99.9
Lunch	124	(66.3)	59.3 – 72.7	154	(77.0)	70.7 – 82.3	71	(73.2)	63.6 – 81.0	24	(63.2)	47.3 – 76.6
Dinner	181	(96.8)	93.2 – 98.5	114	(57.0)	50.1 – 63.7	79	(81.4)	72.6 – 87.9	14	(36.8)	23.4 – 52.7
Inadequate protein intake per meal (<0.4 g/kg/meal)												
Breakfast	144	(77.0)	70.5 – 82.5	188	(94.0)	89.8 – 96.5	88	(90.7)	83.3 – 95.0	37	(97.4)	86.5 – 99.9
Lunch	93	(49.7)	42.6 – 56.8	161	(80.5)	74.5 – 85.4	70	(72.2)	62.5 – 80.1	23	(60.5)	44.7 – 74.4
Dinner	172	(92.0)	87.2 – 95.1	128	(64.0)	57.1 – 70.3	78	(80.4)	71.4 – 87.1	12	(31.6)	19.1 – 47.5
Number of meals per day with ≥30 g protein												
Zero	114	(61.0)	53.8 – 67.7	84	(42.0)	35.4 – 48.9	59	(60.8)	50.9 – 69.9	4	(10.5)	4.2 – 24.1
One	64	(34.2)	27.8 – 41.3	87	(43.5)	36.8 – 50.4	28	(28.9)	20.8 – 38.6	29	(76.3)	60.8 – 87.0
Two or three	9	(4.8)	2.6 – 8.9	29	(14.5)	10.3 – 20.1	10	(10.3)	5.7 – 17.9	5	(13.2)	5.8 – 27.3
Number of meals per day with ≥0.4 g protein/kg												
Zero	77	(41.2)	34.4 – 48.3	97	(48.5)	41.7 – 55.4	56	(57.7)	47.8 – 67.1	3	(7.9)	2.7 – 20.8
One	73	(39.0)	32.3 – 46.2	84	(42.0)	35.4 – 48.9	30	(30.9)	22.6 – 40.7	28	(73.7)	58.0 – 85.0
Two or three	37	(19.8)	14.7 – 26.1	19	(9.5)	6.2 – 14.4	11	(11.3)	6.5 – 19.2	7	(18.4)	9.2 – 33.4

95%CI: 95% confidence intervals; g/kg/d: grams of protein per kilogram of body mass per day; U.S.A.: United States of America.

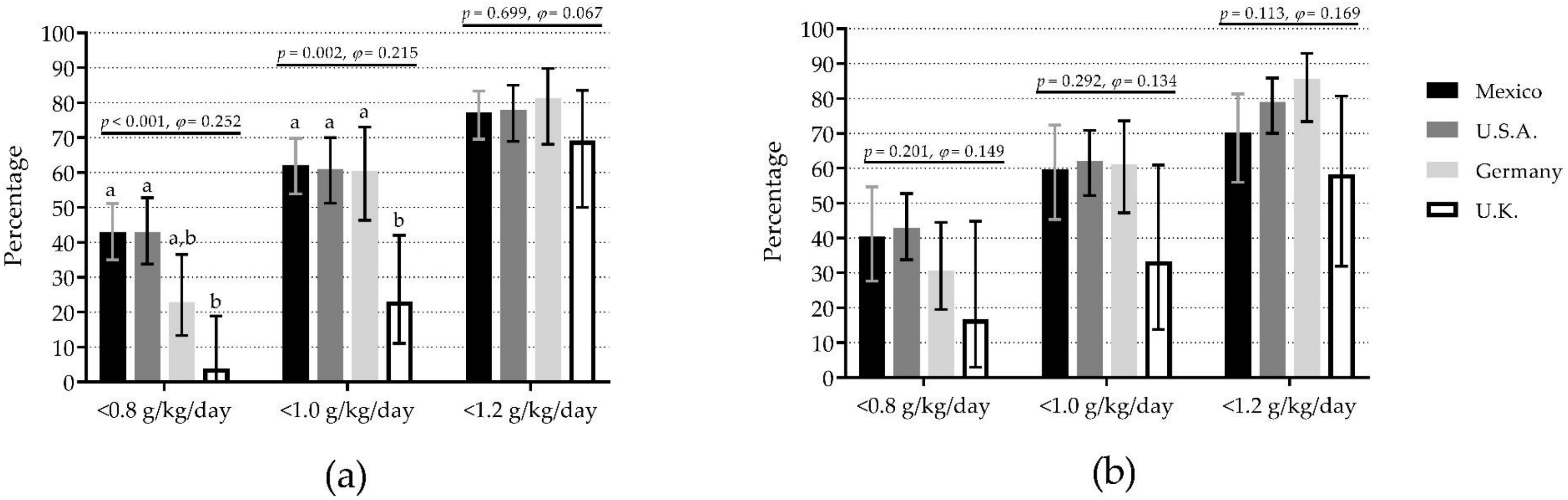

Figure S1. Comparison of inadequate protein intake per day with different cut-points among older adults from four countries in women (**a**) and men (**b**). Bars represent the percentage of inadequate protein intake per day; whiskers represent 95% confidence intervals. *p*-values and φ statistic for comparisons among countries within cut-points (χ^2 test of independence). Bars not sharing a similar letter denote significant differences ($p \leq 0.05$) among countries within cut-points (*t*-test for proportions with Bonferroni correction). g/kg/day: grams of protein per kilogram of body mass per day; U.K.: United Kingdom; U.S.A.: United States of America.

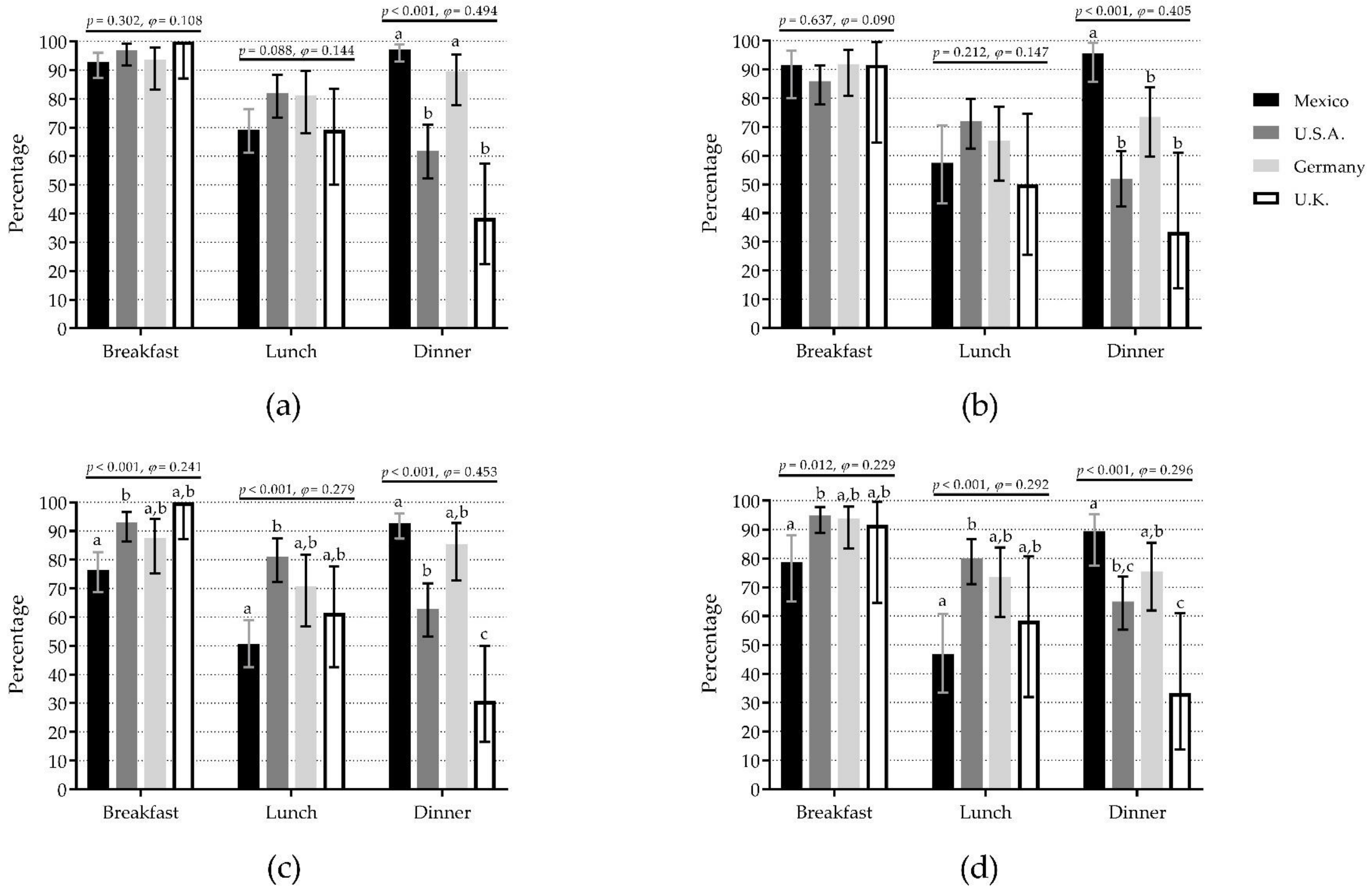

Figure S2. Comparison of inadequate protein intake per meal (breakfast, lunch, dinner) among four countries depending on the protein content for each meal as <30 g/meal (**a, b**) or <0.4 g/kg body mass/meal (**c, d**) in women (**a, c**) and men (**b, d**). Bars represent the percentage of inadequate protein intake per meal; whiskers represent 95% confidence intervals. *p*-values and φ statistic for comparisons among countries within meals (χ^2 test of independence). Bars not sharing a similar letter denote significant differences ($p \leq 0.05$) among countries within meals (*t*-test for proportions with Bonferroni correction). U.K.: United Kingdom; U.S.A.: United States of America.

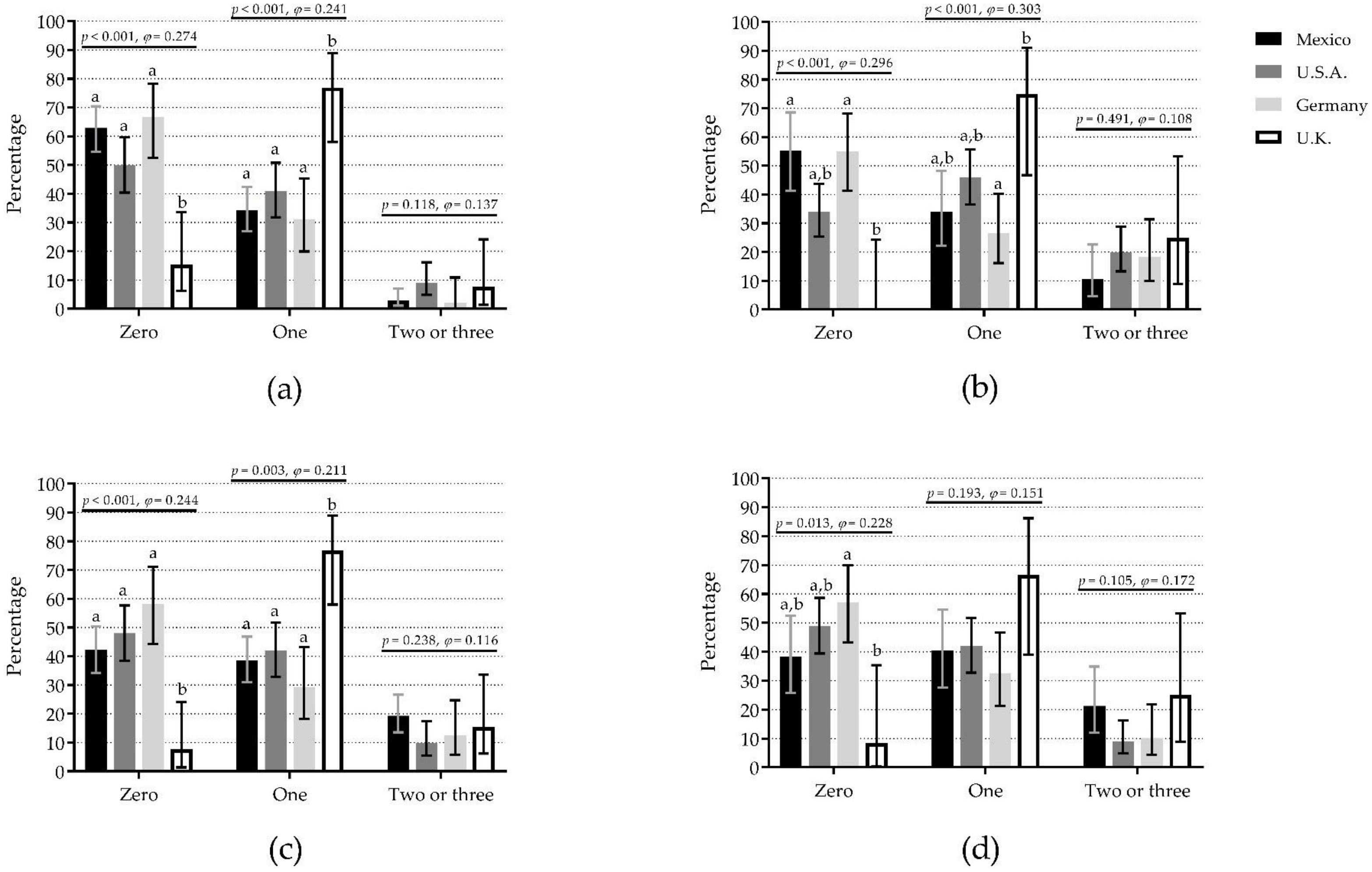

Figure S3. The number of meals per day containing ≥30 g protein (**a, b**) or ≥0.4 g protein/kg body mass (**c, d**), in women (**a, c**) and men (**b, d**) and compared among countries. Bars represent the percentage of participants that reported the number of meals per day (zero, one, two or three) with the mentioned protein content; whiskers represent 95% confidence intervals. *p*-values and φ statistic for comparisons among countries within the number of meals (χ^2 test of independence). Bars not sharing a similar letter denote significant differences ($p \leq 0.05$) among countries within the number of meals (*t*-test for proportions with Bonferroni correction). U.K.: United Kingdom; U.S.A.: United States of America.
